# Phenotypic and Functional Characterization of Peripheral Sensory Neurons derived from Human Embryonic Stem Cells

**DOI:** 10.1038/s41598-017-19093-0

**Published:** 2018-01-12

**Authors:** Abdullah Jawad Alshawaf, Serena Viventi, Wanzhi Qiu, Giovanna D’Abaco, Bryony Nayagam, Michael Erlichster, Gursharan Chana, Ian Everall, Jason Ivanusic, Efstratios Skafidas, Mirella Dottori

**Affiliations:** 1 Centre for Neural Engineering, The University of Melbourne, Melbourne, Australia; 2 Department of Psychiatry, The University of Melbourne, Melbourne, Australia; 3 Department of Physiological Sciences, College of Medicine, Alfaisal University, Riyadh, Saudi Arabia; 4 Department of Biomedical Engineering, The University of Melbourne, Melbourne, Australia; 5 Department of Electrical and Electronic Engineering, The University of Melbourne, Melbourne, Australia; 6 Departments of Audiology and Speech Pathology and Ophthalmology, The University of Melbourne, Melbourne, Australia; 7 Department of Medicine, The University of Melbourne, Royal Melbourne Hospital, Melbourne, Australia; 8 Department of Anatomy and Neuroscience, The University of Melbourne, Melbourne, Australia; 9 Illawarra Health and Medical Research Institute, Centre for Molecular and Medical Bioscience, University of Wollongong, Wollongong, Australia

## Abstract

The dorsal root ganglia (DRG) consist of a multitude of sensory neuronal subtypes that function to relay sensory stimuli, including temperature, pressure, pain and position to the central nervous system. Our knowledge of DRG sensory neurons have been predominantly driven by animal studies and considerably less is known about the human DRG. Human embryonic stem cells (hESC) are valuable resource to help close this gap. Our previous studies reported an efficient system for deriving neural crest and DRG sensory neurons from hESC. Here we show that this differentiation system gives rise to heterogeneous populations of sensory neuronal subtypes as demonstrated by phenotypic and functional analyses. Furthermore, using microelectrode arrays the maturation rate of the hESC-derived sensory neuronal cultures was monitored over 8 weeks in culture, showing their spontaneous firing activities starting at about 12 days post-differentiation and reaching maximum firing at about 6 weeks. These studies are highly valuable for developing an *in vitro* platform to study the diversity of sensory neuronal subtypes found within the human DRG.

## Introduction

Sensory neurons of the dorsal root ganglia (DRG) are a heterogeneous population of neurons that subserve a number of different functions, develop at different times and have diverse central and peripheral projections. DRG neurons may be classically defined on the basis of their sensory modality, electrophysiological properties, axonal diameter and receptor expression profile. This classification includes the broad populations of nociceptors, thermoreceptors, low-threshold mechanoreceptors or proprioceptors^1^. Nociceptors and thermoreceptors are typically small diameter (<30 μm) sensory neurons and have thinly myelinated (Aδ) or unmyelinated (C) axons^1^. Nociceptors respond to noxious mechanical, chemical or thermal stimuli and can be identified by expression of combinations of a number of different markers, including TrkA, TRPV1, calcitonin gene-related peptide (CGRP) and substance P^1^. Some nociceptors, known as polymodal nociceptors, respond to multiple stimuli and many of these express TRPV1. Expression of neuropeptides such as substance P and CGRP is also used to classify nociceptors as peptidergic. In contrast, thermoreceptors respond to changes in temperature that are not considered noxious, and include those that respond to innocuous heating or cooling. Low-threshold mechanoreceptors are typically larger diameter neurons (>50 μm) and have axons that are thickly myelinated (Aβ), although some are smaller myelinated (Aδ) or unmyelinated (C) neurons^1–4^. They innervate the dermal and epidermal regions of the skin and detect low-threshold mechanical stimuli such as vibration, touch and hair deflection. Proprioceptors innervate muscle tissue to provide sensory feedback information on muscle pressure and tension and also have large diameter, myelinated fibres with fast conduction velocities (Aα fibres)^1,3^.

To date, most of the molecular and functional profiling of sensory DRG subclasses has been driven by animal studies and less in known about human DRG neurons. New studies emerging using single cell gene expression profiling of adult rodent DRGs demonstrates further complexity in defining sensory neuronal subtypes and identifying characteristic gene expression profiles and functionalities^4,5^. As various subclasses of DRG neurons are being more comprehensively defined within the rodent it would be valuable to determine whether similar subtypes exist within the human DRG. Human embryonic stem cells (hESC) may provide a useful resource to help address this gap.

Our previous studies described a chemically defined method for deriving neural crest progenitors from hESC, which efficiently gave rise to DRG sensory neurons^6^. Here we report that DRG neurons derived from this method consist of a heterogeneous population of sensory neuronal subtypes. Using microelectrode arrays (MEA), we observe that hESC-derived DRG neurons are functionally mature, showing phenotypic specification and active firing activities within 8 weeks in culture and are responsive to noxious chemical, heat and osmotic swelling stimuli. These studies are highly significant for understanding the development, molecular and functional characteristics of human DRG sensory neurons, which can then be applied for developing therapies to treat peripheral sensory neuropathies.

## Results

### Phenotypic characterization of hESC-derived sensory neuronal subtypes

We previously published an efficient protocol for deriving neural crest from hESC that involves treatment with small molecule inhibitors of activin/nodal (SB431542) and GSK3β (CHIR99021) pathways followed by bone morphogenic protein 2 (BMP2)^6^. This method is similar to other reports describing generation of neural crest from hESC and generates at least 60% SOX10+ neural crest progenitors, which can then differentiate to neural and non-neural crest derivatives^6–8^. Further differentiation of crest progenitors to DRG sensory neurons is achieved by culturing in media supplemented with nerve growth factor (NGF), neurotrophin 3 (NT3) and brain-derived neurotrophic factor (BDNF) that support neural differentiation to all DRG sensory neuronal lineages.

**Figure 1:**
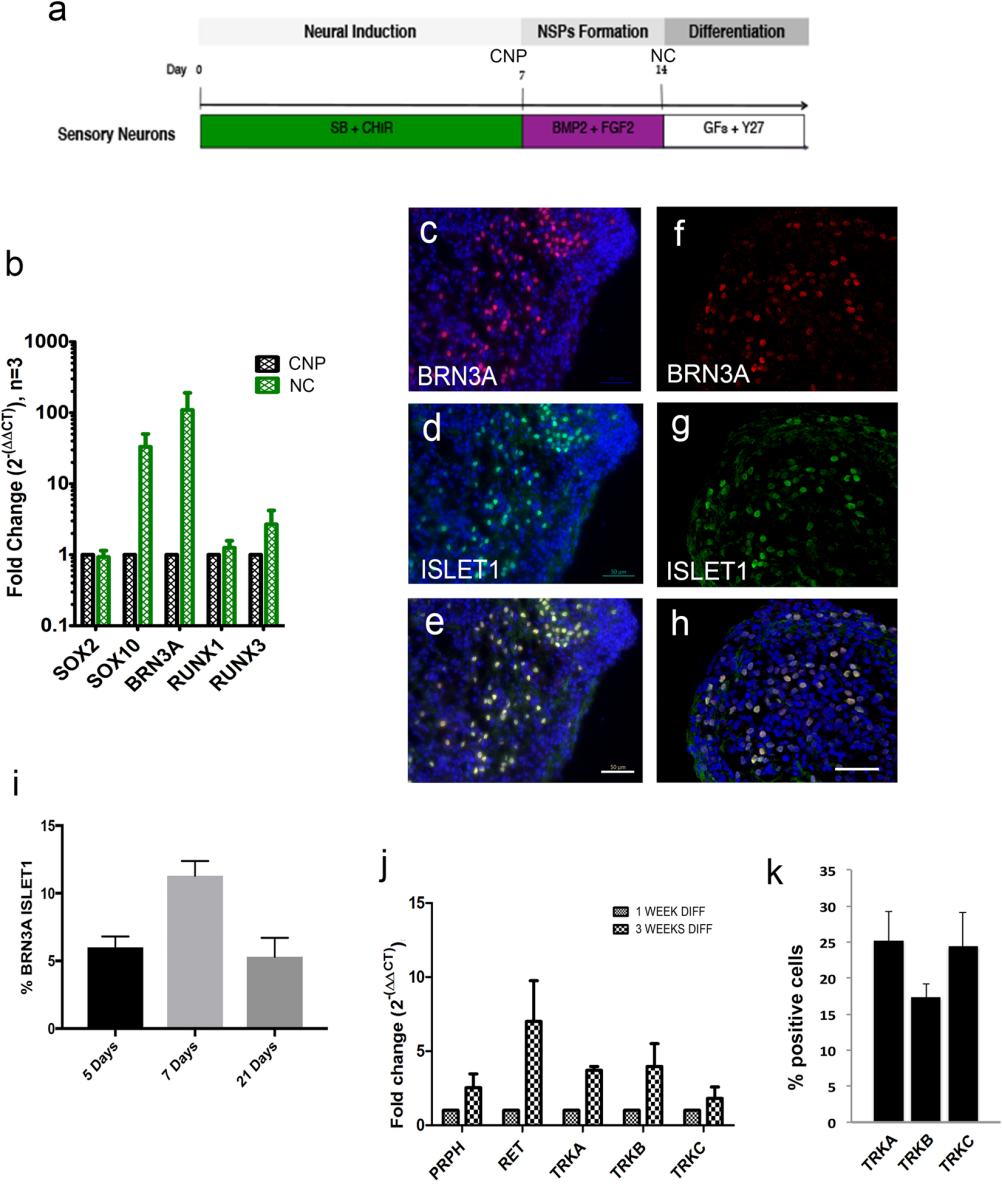
Differentiation of hESC to neural crest and sensory neural progenitors. Schematic diagram outlining stages of neural induction, formation of neural crest neurospheres and differentiation to sensory neurons. (**b**) Q-PCR data shows higher expression of neural crest marker SOX10 and peripheral sensory markers BRN3A, RUNX1 and RUNX3 in neural crest neurospheres relative to caudal neural progenitors. Each experiment has >3 neurospheres and at least 3 independent experiments. (**c**–**f**) Neural crest progenitors differentiated for 5 days (**c**–**e**) and 21 days (**f**,**g**) show co-expression of BRN3A (**c**,**e**,**f** and **h**, red) and ISLET1 (**d**,**e**,**g** and **h**, green). Dapi nuclei are shown in blue (**c**,**d**,**e** and **h**). Scale bars = 50 µm (**e**,**h**). (**i**) Graph showing percentages of neurons co-expressing BRN3A and ISLET1 at 5, 7 and 21 days of differentiation, which are 6.00 ± 0.80 SEM %, 11.03 ± 1.08 SEM % and 5.31 ± 1.39 SEM %, respectively. (**j**) Q-PCR data showing an increase in expression of sensory neuronal markers, Peripherin, RET, TRKA, TRKB and TRKC, at 3 weeks of differentiation relative to 1 week differentiation. N > 3 independent experiments, each experiment has >3 replicate samples. (**k**) Percentage of neurons expressing TRKA (25.20 ± 4.04 SEM), TRKB (17.35 ± 1.82 SEM) and TRK C (24.42 ± 4.75 SEM). Abbreviations: CHIR, CHIR99021; CNP, caudal neural progenitors; GF, growth factors; NC, neural crest; NSPs, neurospheres; SB, SB431542; Y27, Y27632.

Using this protocol, we characterized the types of sensory neurons found within the differentiated cultures. Firstly, up-regulation of DRG progenitor markers was observed at early stages of differentiation, whereby 6.00 ± 0.80% of the cultures show BRN3A and ISLET1 expression after 5 days. At 7 and 21 days of differentiation, BRN3A/ISLET1 co-expression is 11.30 ± 1.08% and 5.31 ± 1.39%, respectively (Fig. 1c–i). From 1 to 3 weeks of differentiation, Q-PCR analyses shows an increase in Peripherin, RET, TRKA, TRKB and TRKC, which are markers associated with subclasses of nociceptor, mechanoreceptor and proprioceptor neurons^1,4^ (Fig. 1j). At 3 weeks differentiation, the proportion of neurons expressing TRK receptors was 25.20 ± 4.04% TRKA, 17.35 ± 1.82% TRKB and 24.42 ± 4.75% TRKC (Fig. 1k). The proportion of neurons expressing TRK receptors is higher relative to BRN3A/ISLET1 co-expressing neurons and it may be that some of the TRK + neurons are not of a DRG sensory phenotype. Of note, however, expression of BRN3A, ISLET1 and TRK receptors in human DRG fetal sensory neurons has not been well described and may or may not be consistent with rodent DRGs.

**Figure 2:**
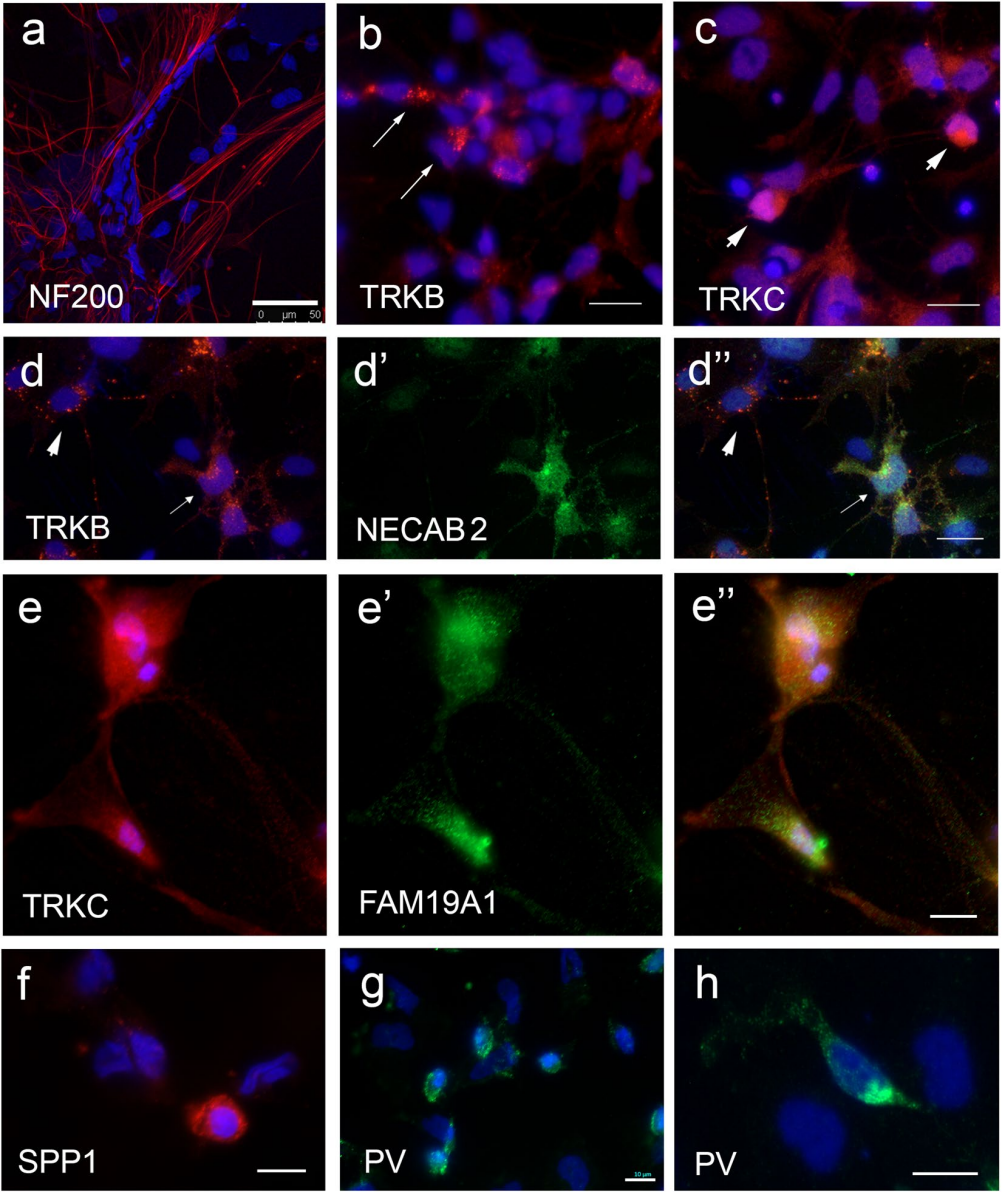
Expression of mechanoreceptor and proprioceptor subtype markers in hESC-derived sensory neuronal cultures. Differentiated neurons were positive for NF200 (**a**, red), TRKB (**b**, red, arrows) or TRKC (**c**, red, arrows). (**d**-d”) A subset of TRKB+ neurons (**d** and d”, red) co-expressed NECAB (d’ and d”, green, arrows). TRKB + and NECAB 2 neurons (**d**-d”, arrowhead) were also observed. (**e**-e”) Neuron co-expressing TRKC (**e** and e” red) and FAM19A1 (e’ and e”, green). (**f**–**h**) Expression of proprioceptors markers, SPP1 (**f**, red) and PV (**g** and **h**, green) in differentiated cultures. Dapi stains of nuclei are shown in blue. Scale bars = 10 µm (**e**,**f**,**g** and **h**), 20 µm (**b**,**c** and **d**) and 50 µm (**a**). Abbreviations: FAM19A1, family with sequence similarity 19 member A1; NECAB 2, N-terminal EF-hand calcium binding protein 2; NF200, Neurofilament 200; PV, parvalbumin; SPP1, osteopontin (secreted phosphoprotein 1).

**Figure 3:**
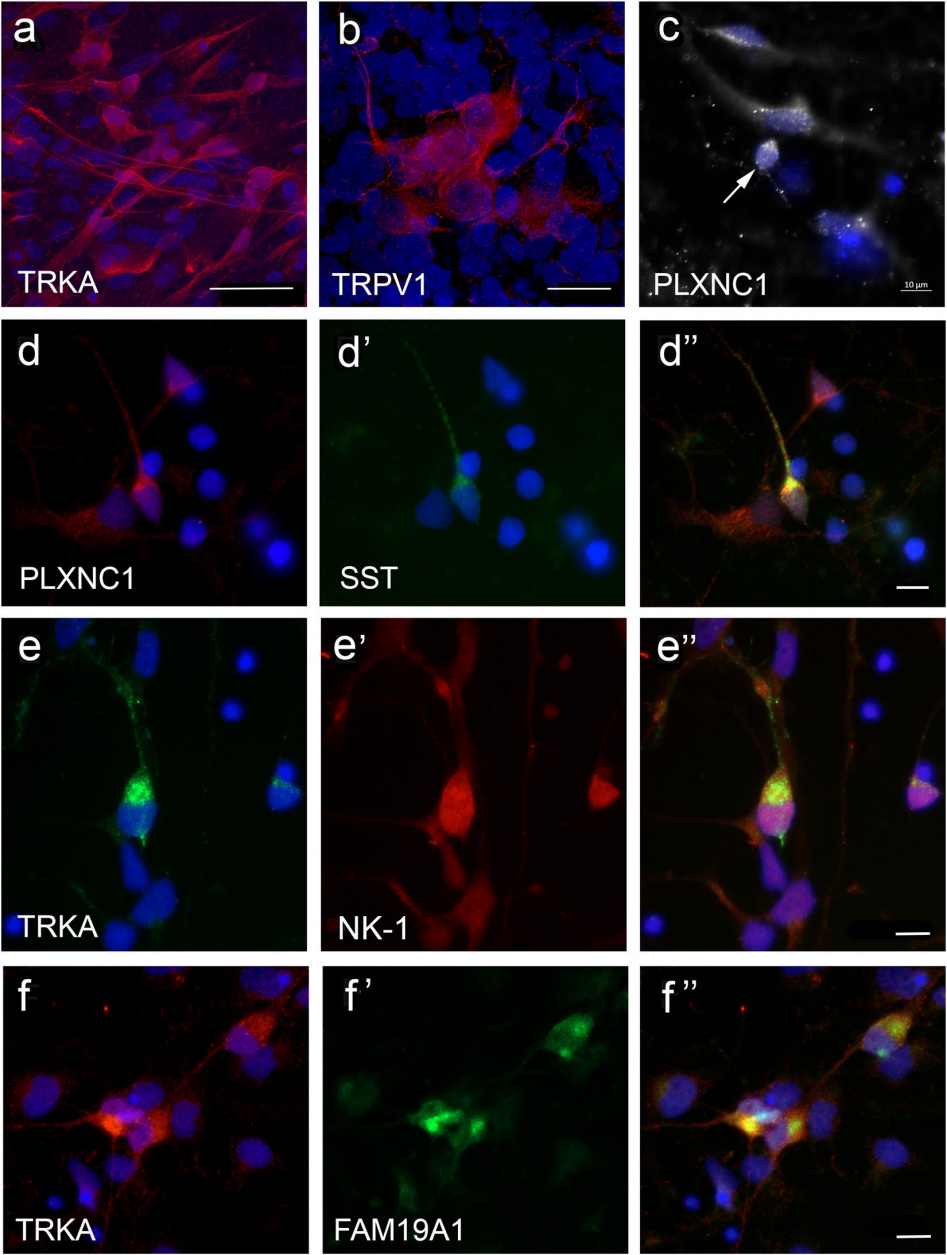
Expression of markers of nociceptor peptidergic and non-peptidergic subtypes in hESC-derived sensory neuronal cultures. (**a**–**c**) Differentiated cultures show clusters of neurons expressing TRKA (**a**, red), TRPV1 (**b**, red) and plexin C1 (**c**, white). (**d**) A subpopulation of plexin C1 (**d** and d”) also expressed SST (d’ and d”, green). (**e**,**f**) Peptidergic neurons showing co-expression of TRKA (**e** and e”, green) and substance P receptor (e’ and e”, red) or TRKA (**f** and f”, red) and FAM19A1 (f’ and f”, green). Dapi stains of nuclei are shown in blue. Scale bars = 10 µm (**c**, d”, e” and f”). Abbreviations: FAM19A1, family with sequence similarity 19 member A1; PLXNC1, plexin C1; SST, somatostatin; NK-1, substance P receptor.

Within the neuronal differentiated cultures, a subset of TRKB+ neurons co-expressed N-terminal EF-hand calcium binding protein 2 (NECAB2) (Fig. 2d-d”) and a subset of TRKC+ neurons co-expressed family with sequence similarity 19 member A1 (FAM19A1) (Fig. 2e-e”). Previous reports profiling rodent DRG neurons suggests that co-expression of these markers are associated with subpopulations of mechanoreceptors^4^. Widespread expression of Neurofilament 200 (NF200) was also observed in the hESC-derived cultures (Fig. 2a), which is classically associated with large diameter mechanoreceptor and proprioceptor neurons^4^. Expression of parvalbumin (PV) and osteopontin (secreted phosphoprotein 1; SPP1), markers of proprioceptor neurons, were also detected (Fig. 2f–h)^4,9,10^. Neuronal clusters expressing nociceptor subtype markers, TRKA and TRPV1, were found within the differentiated cultures (Fig. 3b and c)^4^. Neurons co-expressing Plexin C1 (PLXNC1) and somatostatin (SST) were also detected (Fig. 3d), which may be associated with a subclass of non-peptidergic neurons^4^. Neurons co-expressing TRKA and substance P receptors or TRKA and FAM19A1 were also found in the cultures (Fig. 3e and f), which are characteristic of peptidergic neurons^4^.

Taken together, hESC-derived neuronal cultures consist of heterogeneous populations of sensory neuronal subtypes. The spectrum of sensory neuronal subtypes generated by this differentiation protocol may be applied to model the sensory neurons found within the human DRG.

### Characterizing functional maturation of hESC-derived sensory neuronal cultures using MEAs

To determine the functional maturation rate of hESC-derived sensory neuronal cultures, 1-week-old crest neurospheres were differentiated to sensory neurons on MEAs and recordings were performed twice a week at fixed times for a total of 8 weeks post differentiation. At the end of the experiment, cells were harvested and processed for real time quantitative PCR (Q-PCR) analyses to validate that sensory neurons were present (see Supplementary Figure S1). Measurements of spiking and bursting activities were examined under both non-stimulation and low frequency stimulation conditions. For the latter, MEAs were stimulated twice a week with 2 Hz biphasic voltage pulses for 5 minutes serially on three specific electrodes, which are similar to the stimulation parameters found effective for evoking activity in dissociated cultures of rat cortical neurons^11^.

**Figure 4:**
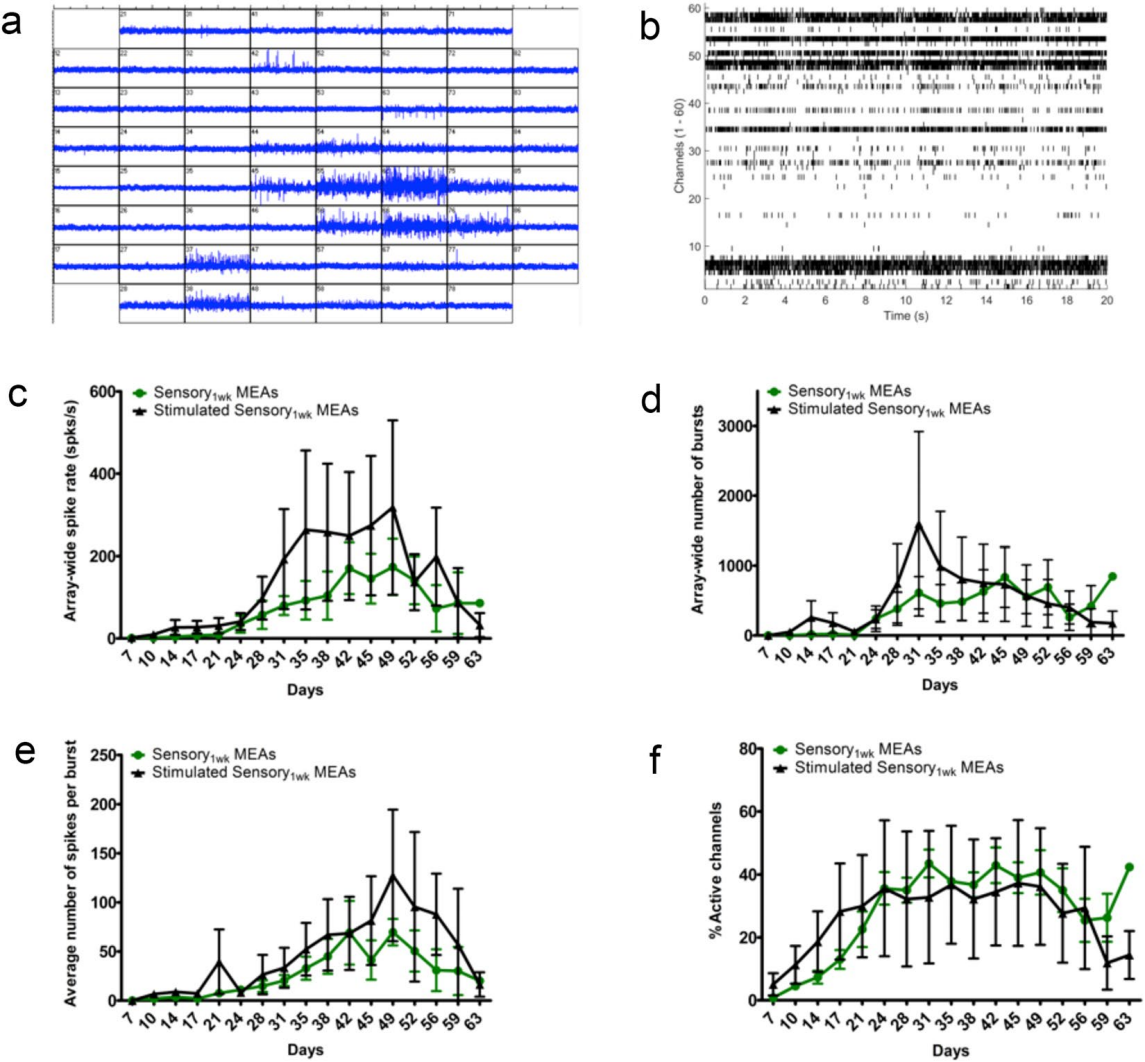
Temporal functional profile of hESC-derived sensory neuronal cultures. (**a**,**b**) Representative snap shot images of a 1 second duration MEA recording (**a**) and a 20 second duration raster plot (**b**) for non-stimulated MEA cultures. (**c**–**g**) Functionality of non-stimulated and stimulated MEA cultures including: measurements of array-wide spike rate (**c)**, array-wide number of bursts (**d**), array-wide number of spikes per burst (**e**) and percentage of active electrodes (**f**) over 60 days were examined on each MEA for each condition; unstimulated MEAs (green) and stimulated MEAs (black). Data for each condition are presented as mean and error bars represent standard error of the mean (SEM).

Spontaneous firing activities were observed in the non-stimulated and stimulated MEAs at 9 ± 1 and 15 ± 8 days post differentiation, respectively (Table 1, also see Supplementary Tables S1 and S2). For non-stimulated MEAs, the maximum array-wide spike rate reached 195.8 ± 63.7 spikes/sec occurring at around 42–49 days post differentiation (Fig. 4c, Table 1). Maximum bursting activity was also observed around this period, being 1021.3 ± 361.5 bursts (Fig. 4d). The maximum average percentage of active channels in non-stimulated conditions was 46.3% ± 4.1 (Fig. 4f, Table 1). The stimulated MEAs showed a similar temporal profile to the non-stimulated MEAs in terms of increasing spiking activity over time (Fig. 4c). Although the array-wide spike rate and bursting activities were higher in the stimulated MEAs, reaching 320.6 ± 211.1 spikes/sec and 1653.3 ± 1300.6 bursts, these values were not significantly different to the non-stimulated MEAs (Fig. 4c and d, Table 1). The maximum percentage of active channels in the stimulated MEAs was 41.2 ± 18.5 (Fig. 4f, Table 1).

**Table 1:** Summary of all non-stimulated and stimulated MEA data presented as mean ± standard error of the mean (SEM).

	Mean ± SEM Non-stimulated MEAs (n = 3)	Mean ± SEM Stimulated MEAs (n = 3)	Mean ± SEM Non-stimulated and Stimulated MEAs (n = 6)
Time when >1 active channel (Days post-differentiation)	9.0 ± 1	15 ± 8	12.0 ± 3.84
Maximum spike rate (Spikes/sec)	195.8 ± 63.7	320.6 ± 211.1	258.2 ± 102.5
Maximum no. of bursts	1021.3 ± 361.5	1653.3 ± 1300.6	1337.3 ± 620
Maximum % of active electrodes	46.3 ± 4.1	41.2 ± 18.5	43.8 ± 8.5

Overall, non-stimulated and stimulated MEAs showed similar profiles in terms of firing activities and maturation rates. Taken together, hESC-derived sensory neuronal cultures start to show spontaneous firing activities as early as 12.0 ± 3.8 days post differentiation (Table 1). Firing activities continue to increase until approximately 6 weeks in culture whereby the maximum array wide spike rate 258.2 ± 102.5 spikes/sec is reached (Table 1). Representative examples of spiking activity evolution for each channel over 8 weeks in non-stimulated and stimulated MEAs are displayed in Supplementary Figure S2. The overall maximum percentage of active channels would reach on average 43.8 ± 8.5%. These findings suggest that hESC-derived sensory neuronal cultures mature to a functionally active state over a relatively short course of differentiation and that low frequency electrical stimulation is not effective in boosting or modifying firing activities.

### Functional characterization of sensory neuronal subtypes

DRG sensory neurons have been broadly classified according to several features including their functions or responses to different types of stimuli. Nociceptors encompass sensory neurons responsive to noxious noxious heat (>42 °C) while other thermosensitive neurons are active at low heat ranges (<40 °C)^12–14^. Mechanoreceptors include static neurons that respond to low threshold mechanical stimuli and populations that have high mechanical sensitivity^13,14^. Phenotypic expression analyses of the hESC-derived sensory neuronal cultures suggest the presence of heterogeneous subtypes. We sought then to couple these analyses with further characterization of sensory neuronal subtypes based on their functional responses to stimuli that they are known to respond to, including heat and hypoosmotic induced membrane stretch. Accordingly, heat and osmolarity assays were applied in two MEAs weekly from week 3 of differentiation for up to a further 5 weeks and spike-sorting analyses were performed to distinguish individual units.

**Figure 5:**
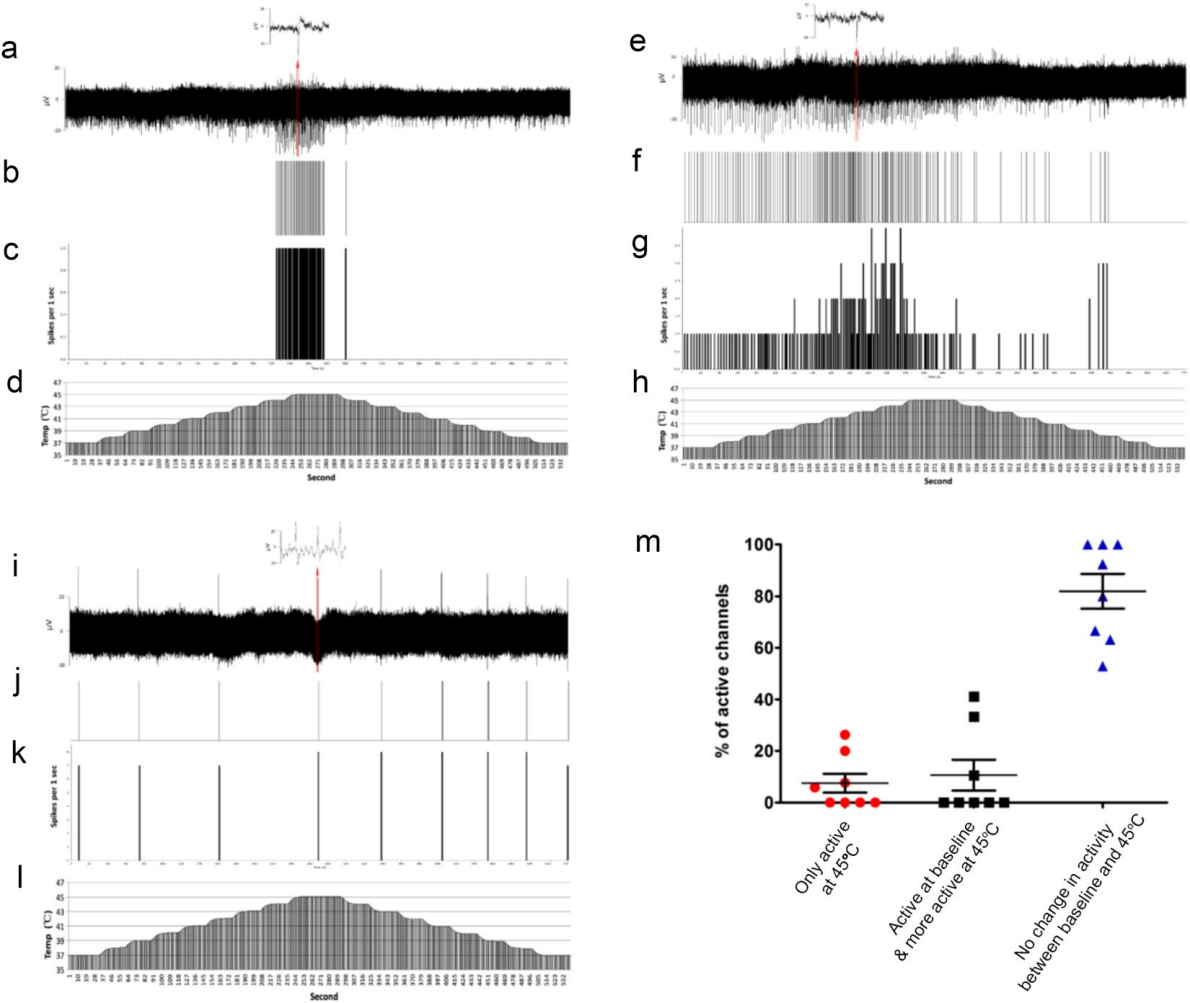
Response of sensory neurons to heat stimuli. (**a**,**e** and **i**) Raw data recordings from 3 different channels showing different spiking responses to changes in temperature over the whole recording (540 seconds), with insets showing an example of spikes in segments of the raw data on the top. (**b**,**f** and **j**) Corresponding raster plot of a single unit isolated from raw data in a, e or i respectively, showing its response to changes in temperature over the whole recording (540 seconds). (**c**,**g** and **k**) Frequency histogram (spikes/1 sec) of a single unit isolated from the raw data in a, e and i respectively. (**d**,**h** and **l**) Line chart showing corresponding temperature changes over the whole recording (540 seconds) for each of a, e and i, where the MEA is incrementally heated (1 °C/30 seconds) from 37 °C to 45 °C and cooled back to 37 °C at the same rate. (**a**–**d**) This single unit showed activity only in response to noxious heat 44 °C-45 °C. (**e**–**h**) This single unit showed an increase in frequency of spike activity in response to changes in temperature from 42 °C-44 °C. (**i**–**l**) This single unit showed no response to changes in temperature. (**m**) Scatter plot grouping channels according to their spiking profile in response to 45 °C relative to baseline 37 °C. Spiking activities from active channels were grouped into 3 categories either no activity at baseline then become active at 45 °C (red circles), active at baseline and showed more activity at 45 °C (black squares) or no change at 45 °C (blue triangles). Data are from weekly recordings of 2 MEAs spanning week 3 to week 8 of differentiation. Mean and standard error of the mean (SEM) are shown.

Thermal MEA assays include two protocols. The first protocol is where incremental temperature increases were applied to the MEA (1 °C/30 seconds) from 37 °C to 45 °C and cooled back to 37 °C. The second protocol is where the MEA was maintained at 37 °C for one minute then instantly heated to 45 °C for another minute and instantly cooled back to 37 °C and recorded further for one minute. Spike sorting analyses of heated MEAs reveal various responses to heat, reflecting heterogeneity of sensory subpopulations in these cultures (Fig. 5). We observed one group of neurons that were exclusively active at 44–45 °C (Fig. 5a–d) and another group of neurons that were active at baseline and showed an increase in spiking activity in response to heating from 42 °C to 44 °C (Fig. 5e–h). We also identified a group that was not responsive to heat as they showed neither increase nor decrease in spiking activity as temperature was changed (Fig. 5i–l). Utilizing the second heat protocol, we then quantified the number of active channels according to the response at 45 °C relative to baseline at 37 °C. Results inform of heterogeneity of these cultures where 81.88% ± 6.70 of active channels were not responsive, 10.63% ± 5.99 showed increased activity at 45 °C relative to baseline at 37 °C, and 7.48% ± 3.63 were only active at 45 °C (Fig. 5m). Those that are exclusively active at temperatures in excess of 44 °C are likely to be C fiber nociceptors that respond to noxious heat, which includes the TRPV1+ polymodal nociceptors^15,16^.

In addition to noxious heat, capsaicin is another stimulant known to activate TRPV1+ polymodal nociceptors^15,16^. Since TRPV1 expression was detected in the hESC-derived sensory neuronal cultures (Fig. 3), we examined their functionality by treating MEA cultures with capsaicin. An increase in bursting activity was observed on some electrodes, but not others, following application of capsaicin to the MEAs (See Supplementary Figure S3).

**Figure 6:**
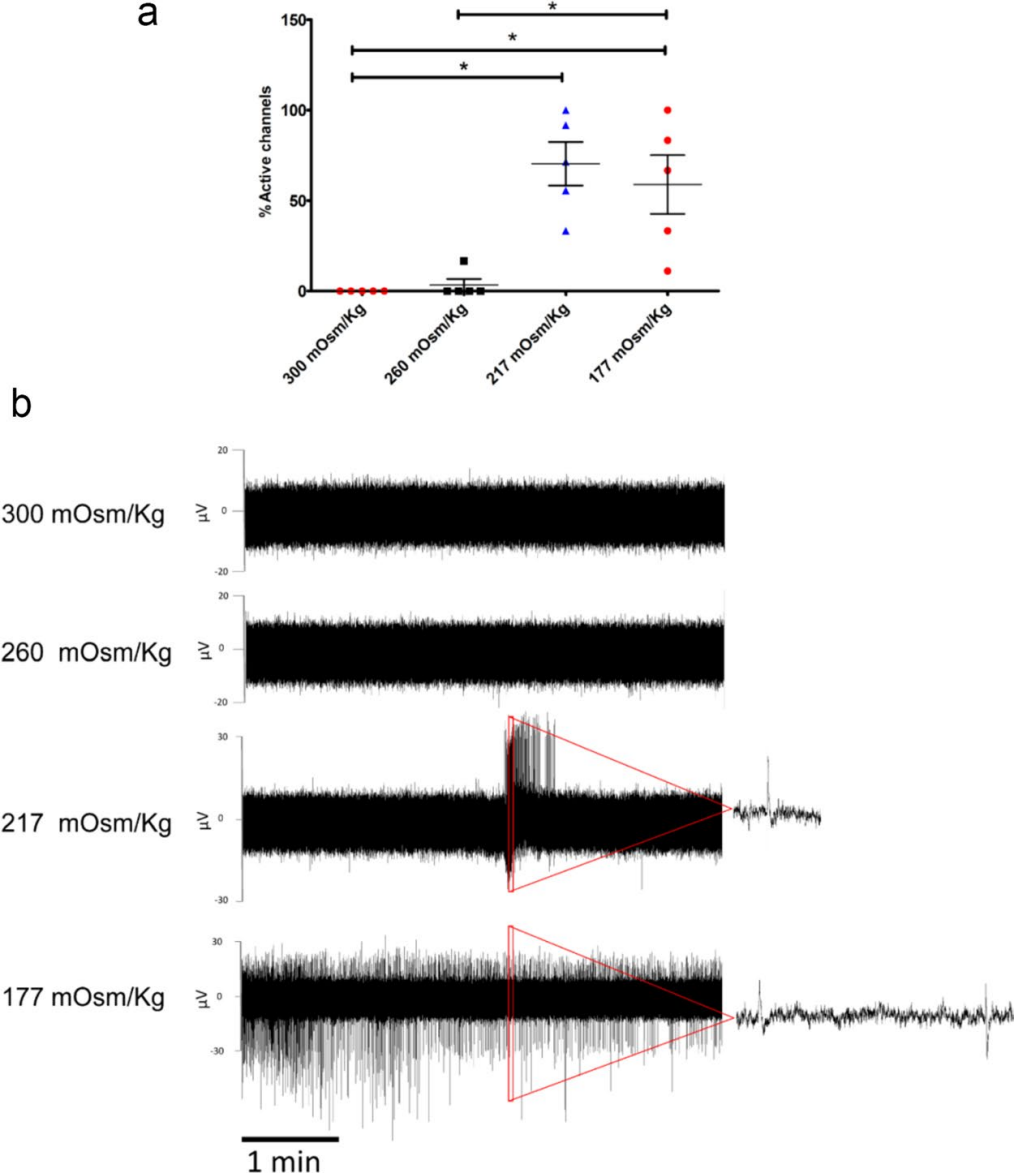
Response of sensory neurons to hypoosmotic stimulus. (**a**) Percentage of active channels in response to reduction in extracellular osmolarity from 300 mOsm/Kg to 177 mOsm/Kg. This data is from weekly recordings of 2 MEAs for a total of 2–3 weeks (5 recording sessions in total). Mean and standard error of the mean (SEM) are shown. Statistical significance was assessed using Krustal-Wallis test with Dunn’s multiple comparison post-test. *P < 0.05 (**b**) Snapshot images of raw data for 5 minute recordings from one channel at each extracellular osmolarity: 300 mOsm/Kg, 260 mOsm/Kg, 217 mOsm/Kg, and 177 mOsm/Kg. Magnified segments of the raw data recording are shown on the right side.

Some nociceptive and mechanoreceptive sensory neurons express mechano-sensitive channels, such as TRPV4 and Piezo 2, that can be activated by changes in membrane tension induced by osmotic swelling^17–19^. To examine whether such neurons may be found within the hESC-derived sensory neuronal cultures, an osmolarity assay was applied to induce membrane stretch (adapted from^20^). MEAs were calibrated to an artificial solution of 300 mOsm/Kg osmolarity and then challenged by employing graded hypoosmotic solutions consecutively as follows; 260 mOsm/Kg, 217 mOsm/Kg and 177 mOsm/Kg. Analyses of data reveal significant effects on the spiking activity of neurons exposed to different hypoosmotic stimuli. Quantification of active channels across solutions with different osmolarities shows a significant increase in the percentage of active channels as osmolarity was decreased, suggesting that hypoosmotic stretch activates mechanically sensitive neurons in the MEAs (Fig. 6a). A representative example of recording is shown in Fig. 6b. These results suggest the presence of functionally mature mechano-sensitive channels in hESC-derived sensory cultures and further highlight heterogeneity of sensory neuronal classes in these cultures.

In summary, these findings further support that the hESC-derived neuronal cultures consist of functionally mature sensory neuronal subpopulations that are not only capable of responding to appropriate stimuli but also demonstrate explicit and heterogeneous responses in line with phenotypic heterogeneity documented in the somatosensory system.

## Discussion

This study is one of the first to describe generation of multiple subclasses of DRG sensory neurons from hESC using one differentiation protocol. The method is based on our previous studies describing an efficient chemically defined system for generating neural crest from hESC^6^. Further differentiation of neural crest progenitors to sensory neurons was in media supplemented with all three neutrotrophic factors, NGF, BDNF and NT3, thereby supporting maturation and survival of most sensory subtypes. The maturation rate of hESC-derived neurons during 60 days in culture was charted using MEAs. Initial spontaneous firing activities were detected as early as week 1–2 post differentiation and reached a maximum average spike rate of 258 spikes/second by week 6. Verification of multiple specific sensory neuronal subtypes was demonstrated according to their responses to heat and hypoosmotic induced stretch. These findings have important implications for establishing *in vitro* models of the human DRG during development and regeneration.

It is difficult to determine the broad proportion of sensory subtypes within the hESC-derived neuronal cultures because they each consist of several subpopulations with overlapping expression of markers. For example, TRKC is expressed in both proprioceptor and some mechanoreceptor subpopulations^4^. Also, there are inconsistencies within the literature that define the gene expression profiles of sensory DRG neuronal subpopulations within the adult rodent^4,5^. For this reason, we can only provide information about the proportion of neurons that express specific receptors, such as TRK receptors, that are generally associated with sensory subpopulations. Confounding this, we observed that the expression of TRK receptors changes during hESC neuronal differentiation, which is consistent with what is observed during DRG development^3,21^. We therefore quantified expression of TRK receptors at three weeks differentiation, which corresponds to when we observe an increase in transcript levels by Q-PCR. At 3 weeks differentiation, the proportion of neurons expressing TRK receptors was approximately 25% TRKA, 17% TRKB and 24% TRKC. Further analyses, such as single cell RNA sequencing, are required to confirm the identity of hESC-derived TRK+ neurons as it may be that some of these are of a non-sensory neuronal phenotype. Despite these issues, what is known about Trk receptor expression in mouse DRG development is that it is dynamic. At E11, mouse DRGs show 20% TrkA, 40% TrkB and 70% TrkC^3,21^. This dramatically changes at E13, which shows 80% TrkA, 8% TrkB and <10% TrkC. Adult human DRGs are reported to have 46% TRKA, 29% TRKB and 24% TRKC^3^. Whilst the proportions of sensory neuronal subtypes found in hESC-derived neuronal cultures needs to be further explored with using multiple markers at different timepoints of differentiation, the molecular and functional data described here suggests the cultures consist of heterogeneous sensory neuronal subtypes. This makes it a useful *in vitro* resource for future studies to perform in-depth phenotypic and functional profiling of specific sensory neuronal subtypes, particularly at the single cell level.

Our results are consistent with previous studies suggesting rapid *in vitro* maturation of hESC-derived sensory neurons. Young *et al*. reported comparability of hESC-derived sensory neurons at day 30 of differentiation to adult human DRG neurons in terms of general gene expression profile and expression of key ion channels^22^. Chambers *et al*. reported three fold faster acquisition of neuronal cell fate of hESC-derived nociceptive sensory neurons than during *in vivo* development and used calcium imaging to demonstrate the capability of these neurons to respond to noxious stimuli^23^. The rapid maturation rate of hESC-derived sensory neurons is advantageous for their potential application in high throughput drug screens for targeting specific peripheral neuropathies. Furthermore, together with using specific sensory neuronal hESC reporter lines, heterogeneous cultures may constitute a more rigorous model for assessing specificity of certain drugs in targeting specific sensory neuronal subtypes.

Electrical stimulation has been studied extensively in DRG neurons for neuronal growth, nerve regeneration and as a therapy for chronic pain^24–26^. Large variations in stimulation parameters exist throughout the literature. Our results suggest that serial low frequency electrical stimulation on three electrodes is not effective in modifying array-wide firing activities of hESC-derived sensory neuronal cultures, although similar electrical stimulation parameters were found to be effective in evoking activity in dissociated cultures of rat cortical neurons^11^. The possibility that different neuronal cell types respond in varying ways to different electrical stimulation parameters cannot be entirely ruled out. Short direct currents rather than alternating currents are found to be effective in promoting neurite outgrowth of chick dissociated DRG neurons^24,25^. As for analgesic use, much higher frequency (>100 Hz) electrical stimulation and stronger voltage pulses exceeding 8 V have been found to be effective in reducing excitability of rat DRG neurons and their propagation of action potentials^26^. Future studies may investigate these parameters using hESC-derived sensory neurons and undertake further characterization of neurite outgrowth, thus offering a human relevant complementary approach for *in vitro* modelling of pain and the identification of potential therapies.

In conclusion, here we show the generation of hESC-derived sensory neuronal cultures, which consist of functionally and neurochemically diverse populations that are consistent with sensory neuronal subtypes found in the DRG. This heterogeneous culture system is significant for creating an *in vitro* model of the human DRG to further study its development, phenotypic and functional properties of human sensory neuronal subtypes and advance regenerative therapies to treat DRG-related disorders.

## Methods

### Maintenance and neural differentiation of hESC

All experiments using hESC were approved by the University of Melbourne Human Ethic Committee (approval number 1545384) and performed in accordance with relevant guidelines and regulations. The H9 (WA-09, WiCell) hESC cell line was cultured feeder free on vitronectin coated plates using MTeSR-1 defined media according to the manufacturer’s instructions (Stem Cell Technologies) and maintained at 37 °C 5% CO2. Colonies were mechanically dissected every 7 days and transferred to freshly coated plates. Cell culture media was changed every day.

For neural induction H9 cells were set up as described by Denham and Dottori^6,27^, with some slight modifications. hESC were mechanically dissected into pieces approximately 0.5 mm in width and transferred to laminin-coated organ culture plates in N2B27 medium containing 1:1 mix of neurobasal medium with DMEM/F12 medium, supplemented with 1% insulin/transferrin/selenium, 1% N2, 1% retinol-free B27, 0.3% glucose, 25U/ml penicillin, and 25 μg/ml streptomycin (Life Technologies/Invitrogen). For induction of sensory neurons, SB431542 (10 μM, Tocris) and GSK3β inhibitor CHIR99021 (3 μM, Tocris) were added to the media for the first 5–7 days to generate caudal neural progenitors, after which they were dissected into 0.5 mm pieces and cultured in suspension in neural basal media supplemented with basic fibroblast growth factor2 (FGF2) (20 ng/ml, Peprotech) and BMP2 (10–50 ng/ml; Peprotech) to form neurospheres consisting of neural crest progenitors. Following 1 week, neurospheres were gently dissociated and plated on poly-δ-lysine and laminin substrates at a density 10–12 neurospheres per MEA dish and/or 3 neurospheres per coverslip in organ culture dish. They were subsequently cultured further for up to 4 weeks in neural basal media supplemented with NGF (10ng/ml, Peprotech), NT3 (10ng/ml, Peprotech), BDNF (10ng/ml, Peprotech) and Y27632 (25 µM, Tocris). Media change was performed every second day. At the end of the experiment samples were either fixed and prepared for immunostaining or processed for Q-PCR analysis.

### Immunostaining

Cell monolayers and neurospheres were fixed in 4% PFA at 4 °C and then washed briefly in PBS. Neurospheres were embedded in Tissue‐Tek OCT compound (Labtek), cut at 15 μm on a cryostat, and sections were placed on superfrost slides. After washing in PBS, sections or culture dishes were permeabilized in 0.1% Triton-X-100 or 0.1% Tween 20 in PBS (PBT) and then blocked in 10% Fetal Calf Serum (FCS) in PBT at room temperature. Samples were then incubated with primary antibody (diluted in the blocking solution) overnight at 4 °C. The following primary antibodies were used: mouse anti-monkey/chicken BRN3A (1:500 Millipore, MAB1585), rabbit anti-mouse/rat/human ISLET1 (1:500, abcam, ab20670), mouse anti-rat NF200 (1:2000, Sigma Aldrich, N0142), goat anti-human TRKA (1:200, R&D Systems, AF175), rabbit anti-human TRPV1 (1:500, Alomone Labs, ACC-030), goat anti-mouse TRKB (1:500, R&D Systems, AF1494), goat anti-mouse/rat TRKC (1:500, R&D Systems, AF1404), rabbit anti-human/mouse NECAB2 (1:1000, Altas Antibodies, HPA013998), rabbit anti-human FAM19A1 (1:100, Altas Antibodies, HPA01307), sheep anti-human/mouse/rat PV (1:500, R&D Systems, AF5058), goat anti-human SPP1 (1:500. R&D Systems, AF1433), mouse anti-human/mouse PLXNC1 (1:200, R&D Systems, MAB3887), rat anti-human SST (Millipore, MAB354) and rabbit anti-rat/mouse/guinea pig Substance P receptor (NK-1) (1:1000, Chemicon International, AB5060). Following three 5‐minute washes in PBT, ALEXA-Fluor secondary antibodies (Life Technologies/Invitrogen) (1:1000 diluted in the blocking solution) were applied for 1 hour at room temperature. All samples were counterstained with 49, 6‐diamidino‐2‐phenylindole (Dapi; 1 ug/ml, SIGMA-Aldrich). Samples were then mounted onto glass slides with 5 µl of moviol aqueous mountant followed by viewing and image capture under Zeiss Axio Observer z1 florescence microscope using ZEN imaging software. Z-stack imaging was performed using Leica SP8 confocal microscope. Images were then reconstructed as an intensity projection using Leica Application Suite X software. Quantification of BRN3A and ISLET1 co-expression was performed relative to DAPI expression using IMAGEJ software as previously described^28^. Quantification of TRKA, TRKB and TRKC expression was performed by cell counting using Adobe Photoshop software. A minimum of 600 cells were counted with n >3 samples per receptor. Some of the primary antibodies were tested in adult rat DRG tissue sections (Supplementary Figure S4). For this procedure, intact DRG tissue was harvested from animals perfused with 4% paraformaldehyde. Fixed tissue was embedded in Tissue-Tek OCT and processed for frozen cryosections, as described above. All experiments using animals were approved by the Florey Institute Animal Ethics Committee (approval number, 15–053) and performed in accordance with relevant guidelines and regulations.

### Gene expression analysis

Cell cultures were harvested and processed for total RNA extraction using PureLink RNA Mini Kit (Life Technologies). Quality of RNA was examined on NanoDrop 2000 Spectrophotometer (Thermo Scientific) with A_260/280_ ratio ranging from 1.98 to 2.05. Up to 2 µg of RNA was used to synthesize first-strand cDNA using SensiFAST cDNA Synthesis Kit (Bioline). Q-PCR reaction and data collection was performed on ViiA7 Real-Time PCR System (Life Technologies) using Universal Master Mix (Applied Biosystems) and raw data were exported to Microsoft Excel for further analysis. TaqMan probes (Life Technologies) used in Q-PCR are displayed in Supplementary Table S3. Each reaction was run in triplicate and contained 4.5 µl (20 ng) of cDNA template in a final reaction volume of 10 µl. Cycling parameters were: 50 °C for 2 minutes, 95 °C for 10 minutes to activate DNA polymerase, then 40 cycles of 95 °C for 15 seconds and 60 °C for 1 minute. ΔCT values were obtained by normalization to the mean of four internal reference genes ELF1 GAPDH, HMBS and TBP. ΔΔCT values were then obtained by normalization to ΔCT values of control samples. Relative gene expression values (fold change) were calculated using the 2^−∆∆CT^ method^29^. Heatmaps of log-transformed gene expression data were generated using the R heatmap.2 function of the gplots package. Default hierarchical clustering and distance functions were used to cluster samples and generate dendrograms. Graphs were generated using GraphPad PRISM software, where data presented as mean with error bars representing standard error of mean (SEM). Statistical significance was assessed using Two-way ANOVA with Bonferroni post-hoc for multiple comparisons.

### MEA data acquisition, electrical stimulation and analysis

Extracellular potentials were recorded using MEA (Multi Channel Systems, Reutlingen, Germany) with a square array of 60 titanium nitride microelectrodes (30 μm diameter, 200 μm inter-electrode distance). The culture temperature was maintained at 37 °C using a TC02 temperature controller (Multi Channel Systems). The electrical signals detected were amplified using a commercial 60-channel amplifier (MEA1060-Inv-BC, Multi Channel Systems). Signals were sampled at 25 kHz using a data acquisition card (MC Card, Multi Channel Systems) for visualization with MC Rack software (Multi Channel Systems) and stored for offline processing using MATLAB. Measurements were performed twice a week. Each measurement session consisted of two blocks of recording each lasting 5 minutes. For stimulated MEAs, each measurement session consisted of 4 sequential blocks of recording, each for 5 minutes: the first block was baseline recording, the second, third and fourth blocks of recording each incorporated stimulation of one fixed electrode: ‘55’, ‘27’ and ‘63’ respectively. The stimulation parameter was 2 Hz biphasic voltage pulses (±800 mV, 200 μsec per phase) and was applied using a STG2004 stimulus generator (Multi Channel Systems)^11^.

Analyses were performed on the first block of recording from each measurement session using MATLAB. Graphs were generated using GraphPad PRISM software where data presented as mean with error bars representing SEM. Statistical significance tests were performed using the Wilcoxon rank sum test, a nonparametric test for equality of population medians of two independent samples^30^.

Our parameters for analysis of MEAs recordings were adopted from previous published studies, which include the following:

#### Definition of an active channel

Spike detection was performed by amplitude thresholding after bandpass filtering the signal (300–6000 Hz). The threshold (Thr) was automatically set to^31^

$${\rm{Thr}}=5{{\rm{\sigma }}}_{{\rm{n}}};{{\rm{\sigma }}}_{{\rm{n}}=}{\rm{median}}\{|{\rm{x}}|/0.6745\}$$

where x is the bandpass-filtered signal and σ_n_ is an estimate of the standard deviation of the background noise^32^. An electrode was regarded as active when its detected spike rate > = 0.1 Hz.

#### Definition of bursts

Bursts were identified according to a method described by Wagenaar *et al*.^33^. Sequences of at least four densely spaced spikes with a maximum inter-spike interval (ISI) of 100 ms were first identified as the core of a burst on individual electrodes. They were extended to include entourage of spikes on the same electrodes with ISIs less than 200 ms.

### Thermal stimulation

Utilizing TC02 temperature controller (Multi Channel Systems), thermal stimulation was conducted in MEAs plated with sensory neurons once every week starting from the third week of differentiation. Temperature was controlled via the software “TCX-Control Program”. Thermal assays include two protocols. The first protocol included two consecutive baseline recording blocks at 37 °C each for 3 minutes followed by a third block where incremental temperature increases were applied to the MEA (1 °C/30 seconds) from 37 °C to 45 °C and cooled back to 37 °C. The second protocol is similar to protocol 1 except in the third block of recording the MEA was maintained at 37 °C for one minute then suddenly heated to 45 °C for another minute and suddenly cooled back to 37 °C and recorded further for one minute. On some occasions, single units were discriminated in the recordings from each channel according to their relative amplitude and duration using Spike Histogram software (LabChart 8, ADInstruments). Graphs were generated using GraphPad Prism where data presented as scatter plot (mean) with error bars representing SEM.

### Hypoosmotic stimulation

Artificial solutions of different osmolarities were prepared as described in Gomis A *et al*. and measured using a cryoscopic osmometer^20^. A total of 4 solutions of different osmolarities were produced: 300 mOsm/Kg, 260 mOsm/Kg, 217 mOsm/Kg and 177 mOsm/Kg. The basic solution contained (in mM): 90 NaCl, 5 KCl, 1.3 MgCl2, 2.4 CaCl2, 10 HEPES, 10 D-glucose, with pH adjusted to 7.4 with NaOH. Mannitol then was added at different amounts to produce different osmolarities including: 300 mOsm/Kg baseline osmolarity solution by adding 1.33 g mannitol, 260 mOsm/Kg hypoosmotic solution by reducing mannitol to 0.70 g, and 217 mOsm/Kg hypoosmotic solution without adding any mannitol. For the 177 mOsm/Kg solution, the basic solution contained only 70 mM NaCl and no mannitol was added.

Osmolarity experiments were conducted in MEAs once every week for 2–3 weeks total starting from the third week of differentiation. Each MEA was calibrated to 300 mOsm/Kg for the first block of recording, 260 mOsm/Kg for the second block of recording, 217 mOsm/Kg for the third block of recording, and 177 mOsm/Kg for the fourth block of recording, with each block of recording lasting for 5 minutes. Graphs were generated using GraphPad Prism and data were presented as a scatter plot (mean) with error bars representing SEM. Statistical significance was performed using non-parametric Krustal-Wallis test with Dunn’s multiple comparison post-test.

### Capsaicin assay

The assay using capsaicin was conducted in sensory MEAs post the third week of differentiation. Two consecutive baseline recordings were conducted each for 3 minutes followed by a third block of recording where capsaicin (2 uM, Sigma-Aldrich) was added at the edge of the well at second 60 and recorded further till second 600.

### Data availability

All data generated or analysed during this study are included in this published article (and its Supplementary Information files).

## Electronic supplementary material

Supplementary Information
