# Supplementary material for: Phenotypic and Functional Characterization of Peripheral Sensory Neurons derived from Human Embryonic Stem Cells
Source: Sci Rep. 2018 Jan 12;8:603. doi: 10.1038/s41598-017-19093-0 (PMC5766621; doi:10.1038/s41598-017-19093-0)
Supplement: Supplementary file 1 — Supplementary Information [file 41598_2017_19093_MOESM1_ESM.pdf]

Phenotypic and Functional Characterization of Peripheral Sensory Neurons derived from Human Embryonic Stem Cells

Abdullah Jawad Alshawaf [1, 2, 3], Serena Viventi [1, 4], Wanzhi Qiu [1, 5], Giovanna D'Abaco [1, 4], Bryony Nayagam [6], Michael Erlichster [1, 7], Gursharan Chana [1, 2, 7], Ian Everall [1, 2], Jason Ivanusic [8], Efstratios Skafidas [1, 2] and **Mirella Dottori** [1, 4, 8, 9 *]

[1] Centre for Neural Engineering, The University of Melbourne, Australia.

[2] Department of Psychiatry, The University of Melbourne, Australia.

[3] Department of Physiological Sciences, College of Medicine, Alfaisal University, Saudi Arabia.

[4] Department of Biomedical Engineering, The University of Melbourne, Australia

[5] Department of Electrical and Electronic Engineering, The University of Melbourne, Australia

[6] Departments of Audiology and Speech Pathology and Ophthalmology, The University of Melbourne, Australia

[7] Department of Medicine, The University of Melbourne, Royal Melbourne Hospital, Australia

[8] Department of Anatomy and Neuroscience, The University of Melbourne, Australia

[9] Illawarra Health and Medical Research Institute, Centre for Molecular and Medical Bioscience, University of Wollongong, Australia

*Corresponding Author
Mirella Dottori
Illawarra Health and Medical Research Institute
Building 32, University of Wollongong, NSW 2522 Australia
Email: mdottori@uow.edu.au

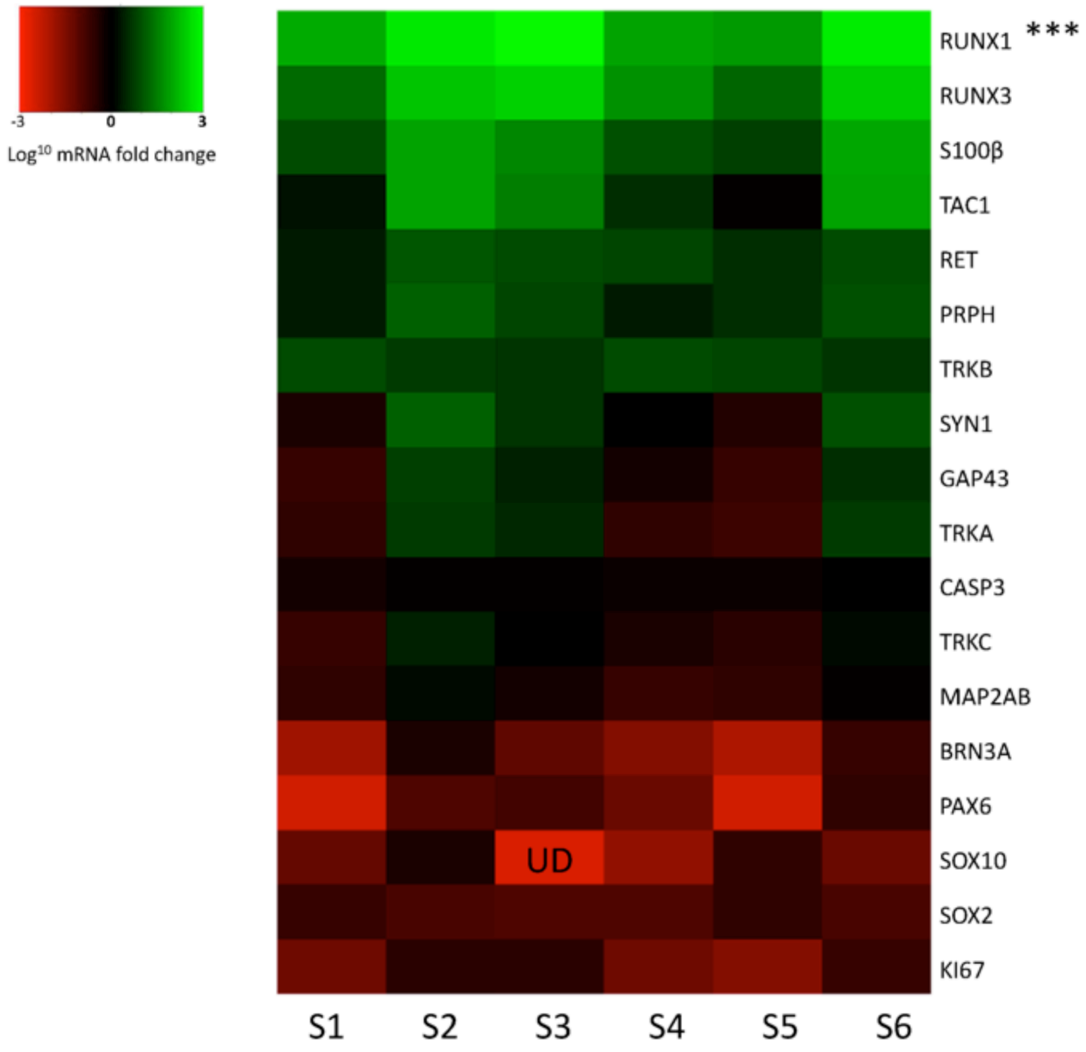

Supplementary Figure S1. **Gene expression data of sensory neurons plated on MEAs.**

Q-PCR was performed on each MEA for each condition; non-stimulated sensory MEAs (S1, S2 and S3), and stimulated sensory MEAs (S4, S5, and S6). The heatmap shows log10 mRNA fold change values relative to 1-week-old neurosphere samples (n=3, > 3 neurospheres per sample); upregulated and downregulated genes are coloured green and red, respectively. Statistical significance was assessed using Two-way ANOVA with a post-hoc Bonferroni multiple comparison, comparing non-stimulated with stimulated sensory MEAs. ***P< 0.001. UD, undetected.

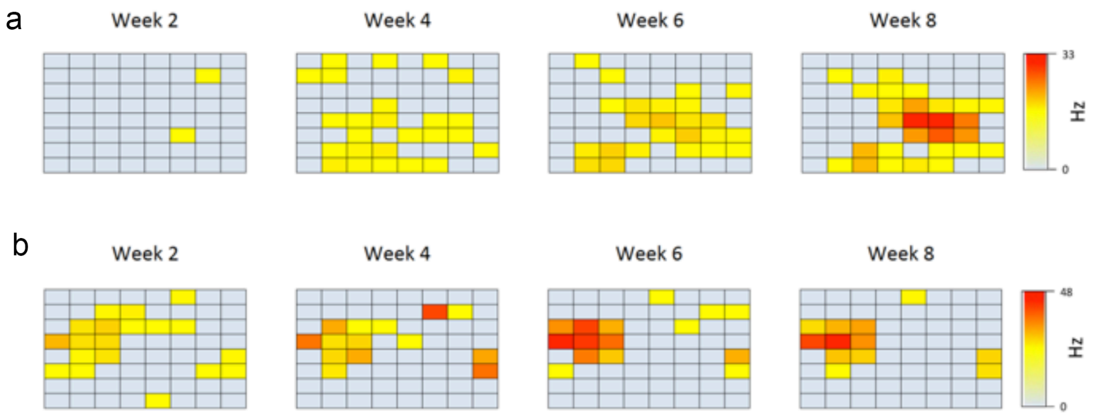

Supplementary Figure S2. **Evolution of spiking activity of sensory MEAs over 8 weeks *in vitro*.**

(a) MEA layout coloured-coded showing spike frequency for each channel in S1 MEA culture at week 2, 4, 6 and 8 post differentiation. (b) MEA layout coloured-coded showing spike frequency for each channel in S6 MEA culture at week 2, 4, 6 and 8 post differentiation. Highest and lowest spike frequencies are coloured red and blue, respectively. Scale bars are in Hz with maximum value of 33 Hz in (a) and 48 Hz in (b).

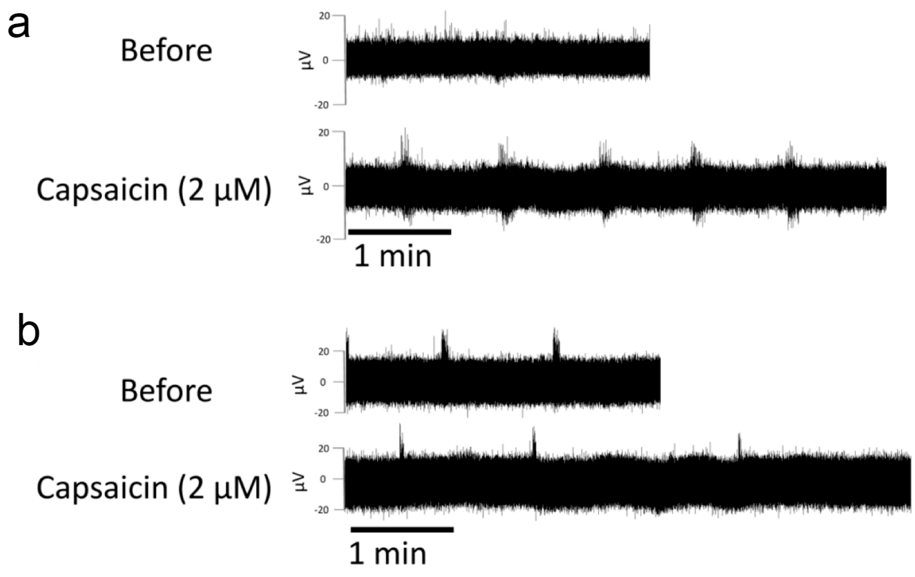

Supplementary Figure S3. Sample electrode recordings before and after application of capsaicin in MEAs.

(a) Snapshot images of a raw data recording before (3 minutes duration) and after (5 minutes duration) application of capsaicin (2 µM). In this example bursting activity is observed in response to application of capsaicin. (b) Snapshot images of a raw data recording before (3 minutes duration) and after (5 minutes duration) application of capsaicin (2 µM). In this example, no change in activity was observed.

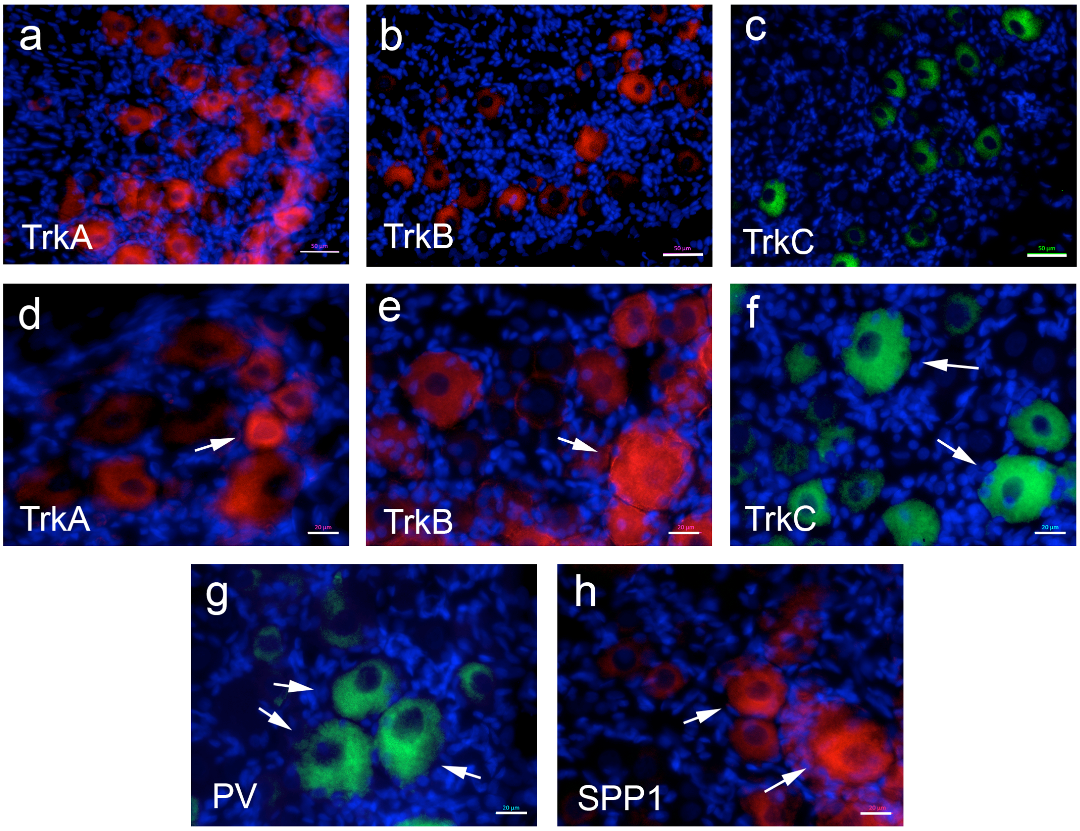

Supplementary Figure S4. Expression of sensory neuronal markers in adult rat DRG tissue.

Immunostaining images of adult rat DRG tissue showing DRG neurons expressing TrkA (a, d, red), TrKB (b, e, red), TrkC (c, f, green), parvalbumin (g, green) and osteopointin (h, red). Dapi stains of nuclei are shown in blue. Scale bars = 50 µm (a-c), 10 µm (d-h). Abbreviations: PV, parvalbumin; SPP1, osteopontin (secreted phosphoprotein 1).

	S1	S2	S3	Mean ± SEM
**Time when > 1 active channel (Days post-differentiation)**	10	7	10	9.0 ± 1
**Maximum spike rate (Spikes/sec)**	276.2	241.0	70.1	195.8 ± 63.7
**Maximum no. of bursts**	1620	1073	371	1021.3 ± 361.5
**Maximum % of active channels**	54.2	40.7	44.1	46.3 ± 4.1

Supplementary Table S1. Data of non-stimulated MEAs; S1, S2 and S3. Mean and standard error of the mean (SEM) of S1-S3 are also shown.

	S4	S5	S6	Mean ± SEM
**Time when > 1 active channel (Days post-differentiation)**	7	31	7	15 ± 8
**Maximum spike rate (Spikes/sec)**	721.8	5.7	234.8	320.6 ± 211.1
**Maximum no. of bursts**	4220	4	736	1653.3 ± 1300.6
**Maximum % of active channels**	76.3	13.6	33.9	41.2 ± 18.5

Supplementary Table S2. Data of stimulated MEAs: S4, S5 and S6. Mean and standard error of the mean (SEM) of S4-S6 are also shown.

Target	Assay ID
BRN3A	Hs00366711_m1
CASP3	Hs00234387_m1
GAP43	Hs00967138_m1
KI67	Hs01032443_m1
MAP2AB	Hs00258900_m1
NTRKA	Hs01021011_m1
NTRKB	Hs00178811_m1
NTRKC	Hs00176797_m1
PAX6	Hs00240871_m1
PRPH	Hs00986946_g1
RET	Hs01120030_m1
RUNX1	Hs01021970_m1
RUNX3	Hs00231709_m1
S100β	Hs00902901_m1
SOX10	Hs00366918_m1
SOX2	Hs01053049_s1
SYN1	Hs00199577_m1
TAC1	Hs00243225_m1
**House Keeping Genes**	
ELF1	Hs00152844_m1
GAPDH	Hs02758991_g1
HMBS	Hs00609296_g1
TBP	Hs00427620_m1

Supplementary Table S3. List of TaqMan probes used in Q-PCR analyses.
